# Stigma and mental health challenges among adolescents living with HIV in selected adolescent-specific antiretroviral therapy clinics in Zomba District, Malawi

**DOI:** 10.1186/s12887-022-03292-4

**Published:** 2022-05-06

**Authors:** Esther C. Kip, Michael Udedi, Kazione Kulisewa, Vivian F. Go, Bradley N. Gaynes

**Affiliations:** 1 Malawi College of Medicine, Private Bag 360, Chichiri, Blantyre 3, Malawi; 2 Malawi Ministry of Health, P.O. Box 30377, Lilongwe 3, Malawi; 3 University of North Carolina, Chapel Hill, USA

**Keywords:** Adherence, Adolescents, AIDS, HIV, Psychosocial experiences, Stigma, Depression

## Abstract

**Background:**

Of the 1.8 million adolescents between the ages of 10 and 19 living with HIV globally in 2020; approximately 1.5 million of these live in sub-Saharan Africa. These adolescents living with HIV (ALHIV) are at higher risk of experiencing mental health problems than those without; in Malawi, 18.9% have a depressive disorder. ALHIV can face numerous psychosocial challenges, but little is known about how ALHIV in Malawi perceive these stressors. Understanding psychosocial challenges of ALHIV is a key step in ensuring good mental health care. The aim of this study was to assess the psychosocial challenges faced by ALHIV attending adolescent-specific ART program in Zomba, Malawi.

**Methods:**

Between April and May 2019, we engaged a purposive sample of ALHIV ages 12–18 (n = 80) in a series of eight focus groups drawing from four Teen Clubs linked to an adolescent-specific ART program. Data were analyzed inductively and deductively to identify themes related to ALHIV psychosocial experiences.

**Results:**

Two themes that emerged from the study include: 1) stigma and discrimination within communities and families; 2) non-adherence to medications. HIV-related stigma was associated with increased psychological distress; physical and emotional/verbal abuse; low social support, isolation, and a feeling of rejection; and risky health behaviors such as medication hiding and non-adherence to ART. Discriminatory actions were manifested in a form of being given separate utensils for their meals and mistreatment at school. Furthermore, some parents did not allow their children to play with the participants out of fear that HIV transmission.

**Conclusions:**

Stigma and discrimination are overlooked potential barriers to HIV treatment and care. If HIV services are to effectively meet ALHIVs' needs, mental health interventions are needed to prevent and manage depression and improve adherence to ART. These findings highlight the crucial need to develop culturally relevant mental interventions aimed at helping ALHIV to cope with these diverse challenges.

## Background

Of the estimated 1.8 million adolescents between the ages of 10 and 19 who were living with HIV globally in 2020, about 1.5 million of these live in sub-Saharan Africa [2]. While 54% of adolescents living with HIV (ALHIV) have been initiated on ART, this group has poorer treatment adherence and viral suppression, and higher mortality rates compared to children and adults [3–8]. The increasing number of ALHIV globally, and their poor virologic outcomes endanger the progress towards eliminating AIDS as a public health threat by 2030 [9].

ALHIV experience physical, social and psychological changes when they transition from adolescence to adulthood and are at increased risk for developing mental health conditions and engaging in health-related risk behaviors [9–13] and for ALHIV, these risks may be even more pronounced. They have the added burden of navigating peer and romantic relationships [7, 12]. These factors contribute to worse HIV outcomes among the adolescents and youth compared to adults [7].

HIV-related stigma and discrimination are major challenges to HIV care [12–18]. Stigma is when someone or people view another person in a negative matter because of a certain illness or attribute. In the context of HIV, stigma is based on the incorrect beliefs and attitudes that devalue people living with HIV [19–23]. ALHIV experience multiple forms of stigma such as “enacted, anticipated, internalized” stigma and are subjected to discrimination within their communities and families both because of their own HIV status and, sometimes, because of their caregivers’ HIV status [14, 15, 18]. ALHIV may directly experience enacted stigma in the form of being stereotyped, excluded, or discriminated against due to their HIV status. Internalized stigma occurs when people believe the negative stereotypes about their identity to be true of the individual and this type of stigma has been related directly to psychological distress for people living with HIV/AIDS resulting in low self-esteem, depression, and helplessness [19, 21–23]. High levels of anxiety, isolation, depression, and suicide ideation have all been connected to stigma and discrimination, all of which have contributed significantly to poor medication adherence and retention in care among ALHIV [4, 7, 13, 17, 18].

Mental health disorders, which are already common among adolescents such as major depressive disorder (referred to as depression from hereon) is a primary contributor to the burden of disease and is estimated to be the leading cause of disability as measured by Years Lost due to Disability (YLDs) amongst adolescents; with suicide ranking as the third most common cause of death [5, 16, 17] and may develop amongst ALHIV for these reasons, and can also be higher prevalence than general population rate. A study in Malawi among ALHIV revealed a depression prevalence rate of 18.9% [5, 16, 17].

In Malawi, nearly two-thirds of the country’s estimated 17.2 million people are under the age of 24 [9]. Youth and adolescents, aged 10–24, account for about 50% of new HIV infections, with prevalence higher among 15–17-year olds [2]. HIV-related stigma, discrimination and mental health challenges are especially critical [9]. Many ALHIV may be orphans, and may be managing concerns about food security, livelihood and household issues in addition to their health [2, 9]. Despite services to support ALHIV, there are many social economic and contextual issues [2, 3, 9, 13] that are not comprehensively addressed by the adolescent specific ART programs, known as Teen Club program in Malawi. For this vulnerable group, little is known about the stigma, discrimination and psychosocial and mental health factors affecting their daily well-being [3–9, 12, 13]. Therefore, to learn more about mental health issues distressing ALHIV, we assessed the psychosocial challenges faced by ALHIV attending an adolescent-specific ART program, Teen Clubs, in Zomba, Malawi.

## Methods

### Study design

We conducted this cross-sectional qualitative study between April and May 2019.

### Study sites

Four study sites were selected from a cluster of 135 Teen Club clinics that provided treatment and care to ALHIV nationally [9]. We chose these sites because they were implementing ALHIV activities and involved a high number of active ALHIV. In addition, we selected an urban (Zomba Central Hospital) and rural (Machinga District Hospital, Likangala and Ntaja health centers) locations to allow for greater transferability of study findings. In Malawi, ALHIV receive care and support in adolescent-specific ART clinics termed “Teen Clubs”. The Teen Club model as a ‘one-stop’ center caters to young people’s medical needs (including sexual and reproductive health), as well as providing social, relational, and psychosocial support [2, 9, 13]. It provides a space for ALHIV to walk in at any time to access counselling and empowers them to let their needs to be known [24–26]. The success of the Teen Club shows the importance of a one-stop center with the availability of mentors and health providers to monitor and protect young people [2, 9, 13, 24].

### Study population

This study engaged ALHIV, who were accessing services from the 4 adolescent-specific ART sites namely, Zomba Central Hospital (Tisungane), Machinga District Hospital, Likangala and Ntaja health centers. Some of the participants were attending boarding schools. They were included in the study if they were: (1) aged between 12 and 18 years; (2) receiving HIV treatment and care services from the selected ART clinics; (3) able to communicate in the local language (Chichewa or Yao); (4) allowed by a consenting parent or guardian to participate; (5) able to give assent and present themselves on the day of the interview.

### Participant recruitment

As part of initial screening process, the research team together with the health care providers (HCPs) contacted ALHIV through their parent or guardian to inform them about the research objectives. Those who accepted the invitation to participate were informed about the purpose of the study and that we sought ethical approval from the College of Medicine Research and Ethics Committee (COMREC). We also explained to them that participating in the study was completely voluntary and that they were free to refuse to answer any question which made them uncomfortable; in addition, that they were free to withdraw from the study at any stage. We also mentioned to them that we were not going to link data from personal narratives to any personal clinical data. We also communicated about confidentiality issues that all participants’ information and records that contained names or other personal identifiers, such as informed consent forms, were to be stored securely in a locked cabinet in areas with access limited to study staff.

### Sampling strategy and sample size

A purposive sampling approach was used to select the participants. The needs of young people vary according to age, sex, class, religion and culture, urban or rural residence, and whether they are in school or out of school, married or unmarried, sexually active or not. In addition, we made every effort to ensure that our sample was gender balanced.

### Data collection

Data collection was coordinated by the Principal Researcher (PR), who worked with one trained Research Assistant (RA) in social science and qualitative data collection. The RA had been trained on the study protocol, interview skills, and research ethics including specific issues related to research with human participants and ALHIV. Before the focus group discussions (FGDs), the interviewers clearly described the study goals and objectives and obtained informed or assent consent forms from each research participant (and from a parent or guardian for adolescents below the age of 18). They also explained how the data would be used and the procedures in place to protect the anonymity and confidentiality of informants. All the FGDs were recorded with a digital audio recorder. The researcher explained the purpose of the audio recorder, how the recordings would be used, where recordings would be stored and when they would be destroyed. The discussions lasted up to one hour. Additionally, we took some notes during the FGDs, and these notes complemented the audio recordings during the transcription process. Honoring existing rules to maintain the confidentiality of Teen Club participants, no photos or videos were used to collect data during these sessions.

### Ethical considerations

We conducted this study in keeping with guidelines related to research involving human subjects. All methods were performed in accordance with the relevant guidelines and regulations. The study protocol and tools were approved by the College of Medicine Research and Ethics Committee (COMREC Ref No. P.01/19/2577). The RA was re-trained on the ethical issues prior to data collection. At the eligibility screening stage, verbal assent from ALHIV and verbal consent from a parent or guardian was obtained. This approach is in line with the National Commission for Science and Technology Sects. 18 and 48 of the Science and Technology Act No.16 of 2003 for Malawi, whereby the parents or guardians and those participants above 18 years had to sign the consent forms and those below 18 years had to sign their assent forms before contributing to the group setting. In this case participants had to identify themselves during the consenting process but not during the discussion. Therefore, informed consent was obtained from all subjects and from their parent and/or legal guardian for participants below 17 years age. In addition, participants were informed that those who might feel uncomfortable while relating their experience of living with HIV, in which case interviewers will stop recording and stop the interview depending on the wishes of the research participants. Furthermore, they were explained that the research team would refer to specific services as needed in case of any emotional distress. The FGDs were conducted without their parents/guardians. To maintain confidentiality, we attribute quotations with only participant’s sex and from which age groups. The research team received training regarding child protection and were asked to sign a Code of Conduct on Child Protection. The study was conducted in accordance with provisions of the study protocol, the Declaration of Helsinki (October 2013) [27], the WHO Handbook for Good Clinical Research Practice (July 2005) [28] and privacy and confidentiality were guaranteed consistent with guidelines for research involving young people [29]. All participants were provided with an equivalent of 10 USD for transportation and time compensation.

### Data analysis

We employed a reflexive thematic analysis technique to analyze data [30–32]. Reflexive thematic analysis is an easily accessible and theoretically flexible interpretative approach to qualitative data analysis that facilitates the identification and analysis of patterns or themes in each data set [31, 32]. The FGDs were transcribed verbatim, and transcripts were entered into NVivo 12 QSR International so that emerging themes associated with perceived psychosocial challenges among ALHIV could be identified. Analysis (thematic coding, generating deductive codes based on interview guide and adding inductive codes iteratively based on emergent themes) [33, 34] was primarily conducted by primary researcher (ECK). Therefore, the results of the analysis represent author one’s interpretations of the data. Prior to familiarizing with the dataset, the primary researcher engaged in the iterative process of reflexivity [30–32]. The first step in the analysis was to read repeatedly through all the transcripts and took notes to obtain an overall understanding of the data in order to gain an in-depth understanding of the context, concepts, codes, and potential themes [30]. As part of code development process, we identified aspects of data that were interesting and could be useful in developing themes [30, 31]. Codes that appeared to be most relevant to the research question had been organized into meaningful themes. Consequently, codes that were prevalent throughout the entire dataset were subsequently informative in the development of our themes. We then reviewed and analyzed coded data to generate themes and sub-themes [30–34]. Subsequently thematic mapping was done followed by reviewing of potential themes whereby we conducted and reviewed the relationships among the data items and codes that informed each theme and sub-theme [32–34]. Finally, we developed a thematic framework, and each theme was defined and named accordingly, and interpretation was done where the relationship between HIV-related stigma themes and categories were established. The overall quality of each theme to ensure that it accurately reflected what was evident in the data was assessed and explored and that when themes were connected, they would provide a rich and detailed account of the ALHIV psychosocial experiences or challenges. Interpretation was done where the relationship between HIV-related stigma themes and categories were established. Direct quotes were obtained and used to explain and describe themes and sub-themes and to ensure that the results accurately conveyed the participants main points.

### Rigor and trustworthiness

Rigor and trustworthiness refer to the extent of confidence qualitative researchers have in their data [32–35]. This is assessed using criteria of credibility, transferability, dependability and conformability [35–42]. To enhance rigor and trustworthiness of the study findings we used the outlined criteria as shown in Table 1.

**Table 1:** Measures to ensure trustworthiness

**STRATEGY**	APPLICABILITY
CREDIBILITY	This refers to confidence and accuracy of the data and the researcher’s interpretation of the data [36-43]. ○ To ensure the credibility of this study, FGDs were used to obtain information from the participants ○ The questions were rephrased, repeated or expanded on different occasions and probing was done where necessary ○ The primary researcher spent adequate time with the participants in order to understand them better and gain insight into the phenomenon under study and their experiences during data collection ○ Each FGD lasted for about an hour ○ Data saturation was ensured ○ Field notes were taken that noted gesture and other non-verbal cues
CONFIRMABILITY	This is the extent to which data collected from the participants was analyzed objectively such that if another researcher examined the same data, they would get the same results [33–40]. ○ FGDs were audio-recorded and transcribed the data verbatim for further analysis to ensure confirmability of the results ○ Illustrative quotes presented in the results to support each theme and sub-theme, or concept also helped the neutrality our findings
DEPENDABILITY	This refers to the stability and consistency of data obtained and the extent to which this data is dependable over time and across conditions [35–42]. ○ To ensure this, we have given a comprehensive description of the study methodology such as study setting, study population and methods used ○ The technique for data collection and data analysis have also been presented in this paper
TRANSFERABILITY	This concept refers to the extent to which the findings from the data can be transferred to other settings or groups (research situation elsewhere) [35–42]. ○ We have provided sufficient information about the demographic characteristics of the participants, the research setting in order to allow others to assess the transferability of the study findings ○ Accordingly, we have provided a rich description of the setting and context where we conducted the study to make our results transferable to other areas ○ To increase the transparency of the interpretation, coding categories are illustrated with direct quotations in the presentation of the results

### Reflexivity/positionality of the researchers

The researchers in this study have strong educational background in public health, social sciences, mental health and qualitative methods, and many years of qualitative research experience in a range of public health issues, including HIV, health and mental health care services. The primary researcher (ECK) is familiar with the ALHIV context, through prior knowledge and interactions with the study population during another study with HCPs in Teen Club Program. Given the strong educational background and research experience of the researchers in the current study, it is believed that the research questions drove the methodology and methods employed to answer the research questions. ECK conducted data analysis and this analysis reflected on her beliefs around the subject before and during the analysis.

## Results

### Demographics

We engaged a sample of ALHIV ages 12–18 (n = 80) in a series of eight focus groups drawing from four adolescent-specific ART programs who fulfilled all eligibility requirements in the four sites. We selected different ALHIV belonging to the age group 12–14 and to the age group 15–18 (Table 2) with the median age of 14 years. Each of the groups were balanced by gender.

**Table 2:** Demographic information

Study Sites and age range	Sex		Median Age	Total Number
	**Male**	**Female**		
Zomba Central Hospital	Frequency	Frequency		
12–14 years	5	5	13 yrs	10
15–18 years	5	5	16 yrs	10
**Total**				**20**
Likangala HC				
12–14 years	5	5	13 yrs	10
15–18 years	5	5	17 yrs	10
**Total**				**20**
Machinga District Hospital				
12–14 years	5	5	13 yrs	10
15–18 years	5	5	17 yrs	10
**Total**				**20**
Ntaja HC				
12–14 years	5	5	13 yrs	10
15–18 years	5	5	17 yrs	10
**Total**				**20**
**Grand Total**				**80**

The findings are discussed along the themes and the sub-categories that were derived from the data. Appropriate direct quotes have been used where relevant to clarify the results and literature is provided to support the reported findings, where appropriate.

### Stigma and discrimination within communities and families

HIV-related stigma, whether internalized, anticipated, or experienced, was reported by the majority of the participants.

#### Enacted stigma linked to fear of HIV transmission

Enacted stigma was uttered as verbal discrimination and shame, and denial, comprising of overt acts of seclusion, which were basically driven by fear of HIV transmission. At home, acts of discrimination included separation of household utensils because of their HIV positive status as one explained:

A*t home for example, it might be that this individual both parents died, and s/he is staying with the relatives and when his/her parents were alive they understood him/her but then these relatives stigmatize and discriminate her/him. They say just give him his/her own plate and they tell him/her that do not mix your plate with the other plates then such an individual end up depressed and you ask yourself why was I born in this world?* (Male participant, older group).

It was also reported that some parents did not allow their children to play with the participants out of fear that HIV transmission would occur.

*When we are playing with our friends, there are some parents who call their children back to the house and they tell them not to play with us because we have this disease. They tell our friends that we will give them HIV* (Female participant, younger group).

#### Challenges with acceptance and discrimination within the family

Some participants mentioned that they were not accepted by family members when they were diagnosed with an HIV positive result.

*There are some parents, once a child has been found HIV positive, they do not accept him/her. They start stigmatizing and discriminating that child and this causes that child to lose hope and not look for the future or progress in life* (Male participant, older group).

*Sometimes when you are at home you can have an argument with the parents/guardians, and they keep on shouting and when you try to go away from them they follow you while shouting and when it is time to eat they will still be shouting and sometimes stigmatizing you* (Female participant, older group)*.*

Most of the participants also complained that they were physically and emotionally abused by their parents, guardians, and peers. The lack of protection by the people closest to them, who not only failed to protect them, but also inflicted abuse was evident.Most of ALHIV had lost one or both parents and they were being taken care of by relatives (uncles, aunts, and grandparents). In the guardian families, ALHIV often experienced physical and emotional violence. Sometimes they were insulted, battered, and discriminated against.

*When you do a small thing, then they shout at you then after that they beat you with sticks so when adolescents have a lot of worries, they get depressed because of those problems and what their parents are doing to them* (Male participant, younger group).

Some participants reported mistreatment by stepmothers, for those who had lost their biological mothers and whose fathers had remarried. Some of the verbal abuse was in a form of name calling and ridiculing them as one said:

*Sometimes when the parents have insulted us by calling us names and we go tell our friends what is happening in our lives, we find that even our own friends start discriminating us. So, at times one just feels that aaaah I am already dead, so I better just go commit suicide, so some people go ahead committing suicide* (Male participant, older group).

#### Peer comparisons

Many participants also discussed how they struggled to come to terms with finding out they were HIV-positive and were even afraid to discuss any HIV-related issues with their parents or guardians as one female participant explained:

*Sometimes it is difficult to accept HIV and even to yourself it is difficult on how to communicate because you ask yourself how you are going to begin asking them and how will they take it because some parents/guardians are very difficult. They even sometimes say “that's your own problem, what do you want me to do then? Go anywhere you want”. So, you even think that even if I ask or tell such people this depression will just be aggravated* (Female participant, older group).

A sense of being different from others was common among participants, both due to their HIV status and the impact HIV has had on their life circumstances. They reported feeling different from their peers because of their illness and having to take medications to survive, and they had internalized negative stereotypes about HIV. They commonly described feeling different from others due to their HIV status, particularly in relation to their parents, guardians, or siblings they lived with and were HIV negative. One said:

*On my side, my main worry is, will I be cured? Because taking ARVs every day is painful; it is painful because sometimes you find that most of the children in the family were born without HIV and you are the only one with HIV and on ARVs and then at times your siblings stigmatize and discriminate you and the parent does not discipline or shout at these siblings then you end up having depression and you ask yourself …can't I be cured from HIV? Also, you ask yourself how you can be liberated from the bondage of taking ARVs every day or if there can be a person who can provide the drugs to cure the disease* (Female participant, older group).

#### Challenges with acceptance and discrimination within the community

Discriminatory acts and attitudes towards the participants, such as verbal abuse was the most frequently reported. Mocking, harassment, derogatory labelling and name calling was frequently used including referring to them as already dead, as shown by some of the following quotes.

#### Bullying from peers in the community and school setting

Most participants expressed concerns about people gossiping about their HIV status. They commonly described being ridiculed/mocked, laughed at, talked about by peers because of their HIV status or the fact that they took ARVs, and this resulted in depression.

*So if you had thought of keeping it as a secret you will find out that many people know and when they see you they start stigmatizing you and this is not good in an individual's life that people should always gossip about you* (Male participant, younger group).

*Some children insult us by calling us names. They say, “you dog, you are surviving because you are on ARVs”. Then we think that it is simply better to die. Sometimes we isolate ourselves and we ask ourselves why are we suffering like that, why do we take ARVs and how did we get this disease?* (Female participant, older group).

*There are also some friends who come to visit at the house and when they see that your parents are not at home, they start discriminating you saying that we are taking ARVs (iwe okumwa mankhwala iwe) so when our parents come back from the field, we tell them* (Male participant , younger group).

The school setting was also mentioned as not conducive environment to some participants as they reported that they were bullied and emotionally abused by their peers.

*When we come to Teen Club on Saturdays sometimes our friends see us here and when we go to school, they start teasing us that they saw us here at the clinic getting ARVs and such things give us some worries and we think that there is nothing good for us in this world* (Female participant, older group).

Additionally, some participants reported that the teachers did not give any punitive measures to the perpetrators as one participant explained:

*There is also lack of protection at school. Sometimes there are naughty students who like abusing us and if they are reported, the teacher would only leniently talk to such people and then the following day they continue and when you go again to the teacher these boys wait for you on the way home and beat you up* (Male participant, younger group).

#### Stigma and discrimination within health settings

The participants described a variety of negative traumatic experiences contributing to their mental health challenges triggered by their relationships and interactions with significant others in their lives specifically the HCPs.

*Sometimes when you go get your viral load results and when they find out that the virus is so high, they don't speak to you in a nice manner, they start shouting at you as if you have committed a crime and yet you did not know how you should take your ARVs* (Male participant, older group).

*Sometimes they test blood for viral load but for us to get to know the results it is difficult and when you want to know and you ask they shout at you about your HIV status and suggest that you should go for viral load test again then you start wondering as to what happened to the blood they took at first for viral load so this is one of the problems I can say about our challenge* (Female Participant, younger group).

Confidentiality issues also were reported as some participants mentioned that they were not free with HCPs because they would reveal their challenges to other people, and also that they would shout and discriminate them as one female participant highlighted:

*Regarding sexual activities, we have some challenges here because there is no proper counsel/advice to help you; young people cannot be free to approach the health care workers here to seek advice and to go ask for any method because of the attitudes you end up being afraid thinking how you are going to approach them to ask for the methods. It is hard that even if we would ask them there is no use because we will not be assisted and as a result, we just avoid to ask them issues on sexual activities* (Female participant, older group).

*There are some challenges because sometimes when we have told our teen Leader s/he also goes around telling some of his colleagues so we get depressed that s/he has also told other people about our problems* (Male participant, younger group).

### Non-adherence to medications

The participants also reported that they had some challenges taking their ARVs due to issues of non-disclosure to other family members and anger to family members when they ill-treated them at home.

#### Covert ARVs use

Stigma-related challenges manifested in participants going to great lengths to mask their HIV status amidst their peers. Risky health behaviors such as covert medication use and non-adherence to ART were also noted.

*Sometimes we have challenges to adhere to our ARVs because at times there are visitors at home who do not know your health status, so it becomes very difficult. You do not feel free to go to the bedroom and take the drugs because when you are taking the drugs from the bottles, they make some sound like “keche...keche” so one thinks....if I go to the bedroom to take my drugs...what are these people going to think. Then you are not free, and you postpone thinking that you will take them after the visitors are gone then you end up forgetting...then you end up not taking the drugs because of the presence of someone* (Male participant, older group).

Medication adherence challenges was also most frequently reported by ALHIV attending boarding schools, where there is lack of privacy in school dormitories. ALHIV reported that they avoided acts that could arouse suspicions about their status, for instance, by avoiding the use of noisy pill bottles, by not taking medications in the presence of their peers, or by not going to the clinic on their appointed date.

*There are some challenges because sometimes you go to a boarding school, and you have a best friend that you go anywhere with him, and you are together all the time but then when it comes to time to take your ARVs and because your best friend is around you fail to take your ARVs at the right time* (Male participant, older group).

#### Psychological abuse at home negatively influencing non-adherence to medications

Some participants indicated that due to mistreatment at home, they felt like stopping to attend the Teen Club program as well as taking their medications (ARVs).

*Sometimes I just feel like quitting coming to Teen Club and quit taking the medications because of how we are treated at home* (Male participant, younger group).

*When you have an argument with the parents and sometimes your siblings, they talk or tease you about your medication then you stop taking the medications because you are depressed that why are you bothering taking these medications. You think I might as well stop taking them and die because I did not ask to have HIV* (Female participant, older group)

## Discussion

To our knowledge this is the first study in Malawi to comprehensively assess stigma and mental health challenges that are faced by ALHIV. Despite advances in HIV care, ALHIV face numerous psychosocial challenges. In this current study the main identified themes included: stigma and discrimination within communities and families and non-adherence to medications.

Experiences of HIV-related stigma and discrimination were reported in multiple formats and spaces by different actors. ALHIV directly experienced enacted stigma in the form of being stereotyped, excluded, or discriminated against due to their HIV status. Stigma and discrimination experienced by ALHIV through the broader community, as well as in school environment are significant barriers to HIV treatment, often leading to negative consequences and poor health outcomes. HIV stigma can be worsened when ALHIV are ostracized or exposed to acts of discrimination and abuse. These findings confirm prior studies [12–15, 17–23, 43–50] in South Africa, Zambia, Tanzania, Ethiopia, Uganda and Kenya which found that ALHIV experienced stigma, discrimination and bullying both at home and school environments. In addition, ALHIV in this current study had some challenges from internalized stigma or self-stigma. ALHIV can conceive negative beliefs about HIV and stigmatize themselves. This form of stigma results in feelings of negative self-image, shame, and guilt because of one’s HIV status [19, 21–23, 43] and this type of stigma has been related directly to psychological distress for people living with HIV/AIDS [19, 21–23]. The possible consequences of internalized stigma include low self-esteem, depression, and helplessness [19, 21]. Internalized stigma undermines treatment adherence and retention in care. This situation can result in poor mental health, less social support, and more HIV-related symptoms in ALHV. The stigma around HIV from peers at school or in their community may also compromise their adherence to medication [7, 17, 18]. Studies in South Africa, Tanzania, Uganda, Zambia, and Kenya have also reported that HIV-related stigma negatively affects the psychological, behavioral, and health outcomes of HIV-infected people [12–15, 17–23, 43–46, 49–55].

Physical, emotional, and verbal abuse as well as depression, isolation, and a feeling of rejection were major challenges among the participants in this current study. Prior studies in Zambia, Tanzania and Uganda have similarly also shown instances of emotional, physical and domestic violence towards ALHIV [2, 3, 49–57]. Studies have further shown that in some instances ALHIV were insulted, battered, discriminated against, and referred to as “walking dead” [2, 3, 33, 57], and that those that had lost their biological mothers had to bear the mistreatment from a stepmother [2, 3, 49–57]. In Zambia, a study showed that young people reported that psychological abuse specifically as having harmed their HIV self-management [57]. Additionally, the researchers in the Zambian study reported that youth in this study described the forms of maltreatment as a common occurrence with detrimental effects on their HIV self-management practices and their mental health [57]. Mental health disorders, such as high levels of anxiety, isolation, depression and suicide ideation, have been reported among ALHIV (10–24 years) and contribute significantly to poor medication adherence and retention in care [49, 57]. Further, these mental health issues have been linked to increased sexual risk behavior [49]. ALHIV deal with multi-dimensional and intersecting stressors such as stigma and discrimination that may increase their risk of mental health challenges.

Negative reception and poor attitudes of HCPs were also noted. ALHIV complained that HCPs spread information about their HIV status. This may undermine ALHIV trust in ART services. A study in Tanzania documented a wide range of discriminatory and stigmatizing practices, and categorized them broadly into neglect, differential treatment, denial of care, testing and disclosing HIV status without consent, and verbal abuse/gossip in health care setting [54]. Similarly, a study in Ethiopia found that common forms of stigma in health facilities were gossiping about patients' status, verbally harassing patients, avoiding and isolating HIV-positive patients, and referring patients for HIV testing without counselling [56]. The Teen Club program is supposedly to be a key resource to provide ALHIV with space to learn and enrich their lives. The support from this program is apparently not only clinical and motivational, but it also encourages adherence to ART and helping to suppress ALHIV’s viral load. HCPs and mentors also are supposed to address ALHIV evolving needs and decision-making processes. However, participants expressed concern with confidentiality issues. Positive and accepting HCPs attitudes towards ALHIV are vital to successful HIV/AIDS care. HCPs with negative attitudes are less likely to spend time in caring for their patients and this tends to reduce the quality of health care they provide. Health care systems are first and foremost human systems. As shown in the findings, ALHIV undergo severe psychological stress and experience loss of hope. They need empathy and mental support to cope with the stressful situation. In this regard, HCPs, can play a vital role in giving care and support. The perception and internalization of HIV-related stigma, coupled with the lack of supportive social relationships from HCPs, can lead to increased substance use, decreased general psychological health, and decreased engagement in healthcare. ALHIV tend to have mental health challenges that impact negatively on adherence and retention in care and health systems that appreciate this reality may be more responsive to it [49, 58]. ALHIV need more reliable care and HCPs have a unique opportunity to provide supportive, comprehensive care, and try to understand their patients.

Non-adherent to medications emerged as a third theme in this study. Participants reported that they had some challenges in taking their ARVs in certain circumstances such as in the presence of other people or in boarding schools. HIV-related stigma has also been associated with increased risky health behaviors such as medication hiding and non-adherence to ART. The fear of stigma and unintentional disclosure can augment medication adherence challenges. Non-disclosure may lead to patients taking their ARV drugs in secret and/or erratically. In accordance with other studies in Malawi, Tanzania, Uganda and Kenya [2, 3, 34, 41, 42], the lack of privacy in dormitories/boarding schools due to overcrowding, and school routines such as early morning classes were disruptive to adolescents’ medication regimens [2, 3, 48, 55, 56]. Concealing their HIV status would negatively impact their adherence to ART because they would have difficulties in getting permission to go and get drug refills. Studies [2, 3, 49–53] have also shown that disclosure and openness were related to resilience and self-efficacy, while silence, secrecy, and stigma contributed to feelings of self-hate, anxiety, hopelessness, and confusion among HIV/AIDS-affected children.

This current study shows a rich patterning of HIV-related stigma, specifically enacted and internalized stigma experiences among ALHIV. Our findings demonstrate the critical need to realize HIV-related stigma and mental health challenges among ALHIV in Teen Club programs in Malawi. The findings indicate that to respond to HIV-related stigma and discrimination effectively among ALHIV, targeted interventions are needed, such as specific HIV education at individual, family, healthcare, and societal levels. Additionally, these findings reinforce existing literature on HIV-related stigma as barriers to adherence to ART. If HIV services are to effectively meet ALHIVs' needs, mental health interventions are needed to prevent and manage depression and improve adherence to ART.

This paper acknowledges that more needs to be done in Malawi to enhance HIV and mental health outcomes among ALHIV. Future studies are needed among ALHIV to explore the psychosocial experiences of those who do not attend these adolescent-specific ART programs (Teen Clubs) and also longitudinal studies to understand stigma, discrimination, and associated sociocultural factors so that mitigating strategies that successfully deal with these issues are designed and implemented. These findings establish the importance of further evaluating the relationship between enacted HIV-related stigma and HIV outcomes among ALHIV but also highlight the need to examine psychological abuse as a separate concept, which is likely to be entangled with their perceptions of self and acceptance of their HIV status. There is also a need for multifaceted concerted efforts at all levels to reduce stigma, distress, discrimination against ALHIV to support better mental health.

### Strengths and limitations

This study has a number of strengths as it was carried out in multiple well-established adolescent-specific ART Teen Club programs in rural and urban settings. All ALHIV were on ARVs. It was conducted during the weekends so that adolescents in boarding schools had an equal opportunity to participate like those in day schools. Furthermore, all ALHIV contacted for the study accepted to participate and so did their guardians indicating that there is minimal non-participation bias and there was more eagerness to share their experiences. To reduce gender bias a similar proportion of male and female adolescents were included in the focus group discussions from each site to ensure gender balance. However, despite the fact that this study produced valuable findings, the generalization of these findings is subject to a number of limitations. First, ALHIV responses on psychosocial experiences might have been under-reported or over-reported leading to social desirability bias. Second, the sample (ALHIV) used has potential for selection bias as it was a sample chosen from the adolescents-specific ART program (Teen Club), which might not be generalizable since we would not be able to include those who did not attend the Teen Club clinic. Finally, we also did not have some information, such as differences in gender responses as well as the ages against each quote within the focus groups to compare the levels of stigma, duration of HIV treatment, and ART regimens used, which might have provided a deeper understanding of our findings. However, we believe that these findings might provide the most detailed exploration yet of this at-risk ALHIV population in Malawi (and sub-Saharan Africa).

## Conclusions

Context specific stigma reduction strategies and psychological support should target multiple levels of influence including intrapersonal, interpersonal, and structural level factors in order to build resilience in ALHIV. These findings highlight the crucial need to develop culturally relevant context specific mental health interventions aimed at helping ALHIV to cope with these diverse challenges. A critical next step is to develop a grant proposal to pilot test a tailored, appropriate evidence-informed group-therapy interventions [26] that can be integrated into the routine care of ALHIV in ART clinics, but also at family and community levels to reduce the stressors; support them moving forward with fewer mental health symptoms, improved relationships, and better medication adherence [59].

## Data Availability

Data supporting the results are available from the authors upon request and with permission from College of Medicine in Malawi.

## Abbreviations

AIDS: Acquired Immune Deficiency Syndrome
ALHIV: Adolescents Living with HIV
ART: Antiretroviral Therapy
ARVs: Antiretroviral drugs
COMREC: College of Medicine Research and Ethics Committee
FGDs: Focus Group Discussions
HCPs: Health Care Providers
HIV: Human Immuno-Deficiency Virus
PR: Principal Investigator
RA: Research Assistant
TC: Teen Club
USD: United States Dollar
YLD: Years Lost to Disability
